# News Items

**Published:** 1885-09

**Authors:** 


### NEWS ITEMS.

The second meeting of the Committee of the American Medical Association, on organization of the Ninth International Medical Congress, will occur in New York on September 3d. The Congress will occur in Washington, D. C., in 1887.

Dr. John Ten Broek, of Paris, Ill., died on August 8, 1885, aged 77 years. He was one of the oldest practitioners of medicine in the State.

The American Dermatological Association held its Ninth Annual Meeting at Greenwich, Conn., on August 26th, 27th and 28th.

Professor Henry B. Sands has resigned his chair of Practice of Surgery in the College of Physicians and Surgeons of New York.

Menthol in twenty per centum solution is recommended as a local anæsthetic for mucous surfaces. It is said to be a good substitute for the hydrochlorate of cocaine, and is less expensive.

The Detroit Medical College and the Michigan College of Medicine, of Detroit, have been consolidated under the name of Detroit College of Medicine.

St. Paul and Minneapolis, Minnesota, each, ~~has~~ have a medical college now.

A ROYAL PHYSICIAN.—Prince Ludwig Ferdinand of Bavaria, who is said to be a son-in-law of ex-Queen Isabella of Spain, and received the degree of Doctor of Medicine at Munich recently, is now engaged in the practice of medicine at Nymphenburg in Bavaria.

Dr. Oliver Wendell Holmes has just passed his seventy-sixth birthday.

The new building for the College of Physicians and Surgeons, of New York, will be located opposite the Roosevelt Hospital.

THE THIRD ANNUAL MEETING OF THE AMERICAN RHINOLOGICAL ASSOCIATION will be held at Lexington, Ky., October 6th, 1885. Papers and Discussions will be devoted exclusively to the Diseases of the Nasal Passages and their sequences.

